# Energetic and Exergetic Analysis of a Transcritical N_2_O Refrigeration Cycle with an Expander

**DOI:** 10.3390/e20010031

**Published:** 2018-01-18

**Authors:** Ze Zhang, Yu Hou, Francis A. Kulacki

**Affiliations:** 1 State Key Laboratory of Multiphase Flow in Power Engineering, Xi’an Jiaotong University, Xi’an 710049, China; 2 Department of Mechanical Engineering, University of Minnesota, Minneapolis, MN 55455, USA

**Keywords:** N_2_O, transcritical refrigeration cycle, COP, exergy analysis

## Abstract

Comparative energy and exergy investigations are reported for a transcritical N_2_O refrigeration cycle with a throttling valve or with an expander when the gas cooler exit temperature varies from 30 to 55 °C and the evaporating temperature varies from −40 to 10 °C. The system performance is also compared with that of similar cycles using CO_2_. Results show that the N_2_O expander cycle exhibits a larger maximum cooling coefficient of performance (COP) and lower optimum discharge pressure than that of the CO_2_ expander cycle and N_2_O throttling valve cycle. It is found that in the N_2_O throttling valve cycle, the irreversibility of the throttling valve is maximum and the exergy losses of the gas cooler and compressor are ordered second and third, respectively. In the N_2_O expander cycle, the largest exergy loss occurs in the gas cooler, followed by the compressor and the expander. Compared with the CO_2_ expander cycle and N_2_O throttling valve cycle, the N_2_O expander cycle has the smallest component-specific exergy loss and the highest exergy efficiency at the same operating conditions and at the optimum discharge pressure. It is also proven that the maximum COP and the maximum exergy efficiency cannot be obtained at the same time for the investigated cycles.

## 1. Introduction

Chlorofluorocarbon (CFC) and hydrofluorocarbon (HFC) refrigerants are now known to be sources of environmental damage: global warming, ozone depletion, greenhouse gas emissions, and air pollution. Consequently, the Montreal protocol countries [1] have agreed to gradually replace CFCs and HFCs with new refrigerants. Owing to their zero ozone depletion potential (ODP) and low global warming potential (GWP), several natural refrigerants such as carbon dioxide, nitrous oxide, air, water, ammonia, and propane have received increasing attention as future refrigerants [2]. As an earliest natural refrigerant, carbon dioxide-based systems have already gained generally wide acceptance due to their good properties, and CO_2_ transcritical refrigeration technology has been widely applied in vehicles, water heaters, heat pumps, and low-temperature cascade refrigerators, and so on. However, the potential of nitrous oxide as a refrigerant is yet to be fully explored. 

As shown in Table 1, nitrous oxide (N_2_O) and carbon dioxide (CO_2_) have similar properties in terms of critical temperature, critical pressure, and molecular weight [3]. The working temperature range of a refrigerant depends on the triple point temperature of the refrigerant. N_2_O can be used down to an evaporating temperature of −90.82 °C. On the other hand, CO_2_ can be used down to an evaporating temperature of −56.56 °C, and a lower temperature cannot be achieved. The other virtue of N_2_O is that the toxicity of N_2_O is more favorable compared to CO_2_. Similar to carbon dioxide, nitrous oxide is non-flammable. Although the GWP of N_2_O is significantly higher than that of CO_2_, its value still falls under the low GWP classification according to a report from the United Nations Environment Programme [4].

Owing to a high throttling loss, the thermal efficiency of the basic transcritical CO_2_ cycle is lower than that of the conventional vapor compression refrigeration cycle, and this issue has been cited to be an area where developments are required [5]. Some modifications of the basic cycle have been tried: using an ejector to replace the throttling valve, using two-stage compression systems, using a mechanical sub-cooling system, using parallel compression, using cascade systems, and introducing internal heat exchangers [6,7,8,9,10,11,12,13,14]. The expander is often employed in refrigeration systems to improve the cycle performance by replacing the throttling valve. The applications and performance analysis of transcritical CO_2_ cycles with an expander have been extensively studied [15,16,17,18].

Compared with investigations of CO_2_ cycles, research on refrigeration systems using N_2_O as the refrigerant are relatively scarce in the open literature. Kruse and Russmann [3] studied a cascade system with a transcritical CO_2_ topping cycle and an N_2_O bottoming cycle. Bhattacharyya et al. [19] modeled and analyzed a similar N_2_O–CO_2_ cascade system. Sarkar and Bhattacharyya [20] found that the system energetic and exergetic performance of a transcritical N_2_O cycle performs better than that of a transcritical CO_2_ cycle. Agrawal et al. [21] introduced a novel two-stage transcritical N_2_O cycle and compared it with a similar cycle configuration of CO_2_.

In this paper, a comparative energy and exergy analysis is performed on a transcritical N_2_O refrigeration cycle with a throttling valve and with an expander. Effects of the evaporating and gas cooler exit temperatures on the COP and exergy loss are investigated. The results are also compared with CO_2_ cycles that have the same configuration.

## 2. System Description

**Figure 1:**
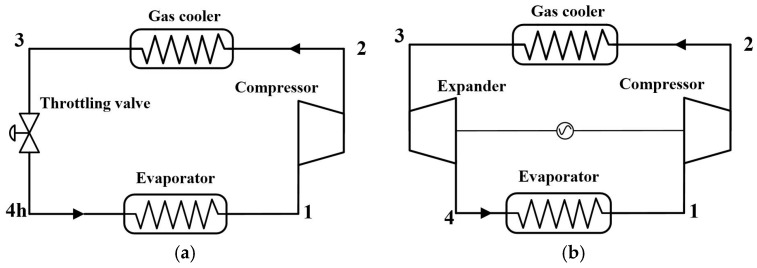
Schematic of the throttling valve cycle (TVC) and the expander cycle (EC). (**a**) Throttling valve cycle and (**b**) expander cycle.

Figure 1 shows the schematic of the transcritical N_2_O refrigeration cycle with a throttling valve and an expander. The cycle consists of a compressor, a gas cooler, a throttling valve or an expander, and an evaporator. Figure 1a shows the throttling valve cycle and Figure 1b shows the expander cycle. 

**Figure 2:**
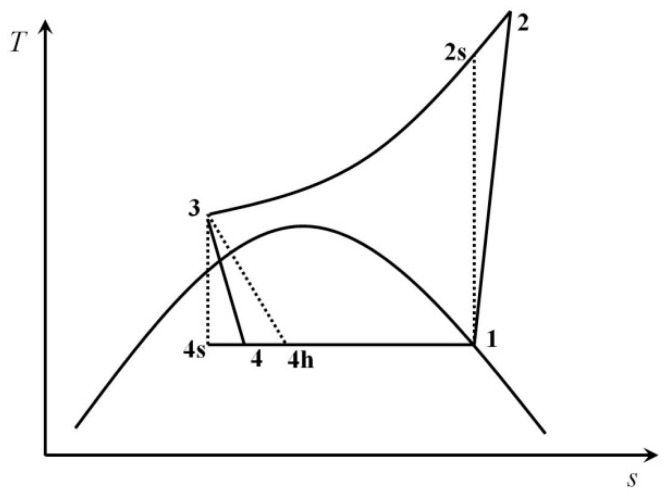
Temperature–entropy diagram of two cycles: the throttling valve cycle (1–2–3–4h–1) and the expander cycle (1–2–3–4–1).

Figure 2 shows the temperature–entropy diagrams of these two cycles. At first, the gas refrigerant outflowing from the evaporator is pressurized by the compressor (1–2) and then cooled in the gas cooler. The working fluid flows through the expansion device (the throttling valve or the expander in this case) in which its pressure and temperature drop significantly (3–4). Then, the gas–liquid refrigerant mixture absorbs heat in the evaporator (4–1).

In the T–s diagram, the process 1–2s is isentropic compression. The process 3–4s is isentropic expansion. The line 1–2s–3–4s–1 shows the ideal refrigeration cycle with an expander. The process 1–2 is the actual compression. The process 3–4 is the actual expansion of the expander. The line 1–2–3–4–1 represents the actual refrigeration cycle with an expander. The process 3–4h is the actual expansion of the throttling valve. The line 1–2–3–4h–1 shows the actual refrigeration cycle with a throttling valve. 

## 3. Thermodynamic Modeling

The system has been modeled based on the energy balance and exergy balance of the individual components of the system under steady-state operation. The main assumptions of the analysis are: (1) the pressure loss and the heat loss are negligible; (2) the working fluid status at state 1 is saturated vapor; (3) the mechanical transmission efficiency between the expander and the compressor has a given value; (4) the isentropic efficiencies of the expander and compressor have a given value and are not affected by operating conditions; (5) the friction loss and the clearance loss of the compressor and the expander are not taken into account; (6) the work output of the expander is used to offset the power required by the compressor.

### 3.1. Energy Analysis

Energy balance equations for the throttling valve cycle and the expander cycle are shown in Table 2.

In Table 2, *w_com_* is the work input to the compressor, *η_com,is_* is the isentropic efficiency of the compressor, *w_exp_* is the output work for the expansion process, *η_exp,is_* is the isentropic efficiency of the expander, *w_exp__,com_* is the work from the expander input to the compressor, *η_mt_* is the mechanical transmission efficiency between the compressor and the expander, *w_com__,elec_* is the electric power input into the compressor.

### 3.2. Exergy Analysis

The main assumptions of the exergy analysis are that the working fluid kinetic energy, internal energy, and their changes are not taken into account. Exergy balance equations for the throttling valve cycle and the expander cycle are shown in Table 3. Exergy losses for the compressor, gas cooler, expander/throttle valve, and evaporator are given. In exergy analysis, the environment temperature T_0_ = 303 K. The refrigerated object temperature *T_r_* is 5 °C higher than the corresponding evaporating temperature.

Therefore, the total exergy loss of the system is

*I_tot_ = I_com_ + I_gc_ + I_exp_ (I_tv_) + I_eva_*. (1)

The exergy efficiency of the refrigeration cycle with a throttling valve is

*ε_tv_ = 1 − I_tot_/w_com_*, (2)

and the exergy efficiency of the refrigeration cycle with an expander is

*ε_exp_ = 1 − I_tot_/w_com__,elec_*. (3)

### 3.3. Parameter Determination

All cycles are simulated within a wide range of operating conditions. The range and assumptions of parameters are as follows:

(1) The compressor has an isentropic efficiency of 75%;
(2) The expander has an isentropic efficiency of 65%;
(3) The mechanical transmission efficiency between the compressor and the expander is 85%;
(4) The outlet temperature of the gas cooler is 30 °C ≤ *t_gc_* ≤ 55 °C;
(5) The evaporating temperature is −40 °C ≤ *t_eva_* ≤ 10 °C.

Based on the above equations and assumptions, simulation programs are developed to evaluate the effect of the expander in the transcritical N_2_O refrigeration cycle. The specific exergy loss in each component is calculated and also expressed as the ratio of the partial exergy loss to the total exergy loss. A performance comparison is also conducted in the similar transcritical cycle using CO_2_ as the refrigerant. The N_2_O property data were obtained from REFPROP [22].

## 4. Results and Discussion

**Figure 3:**
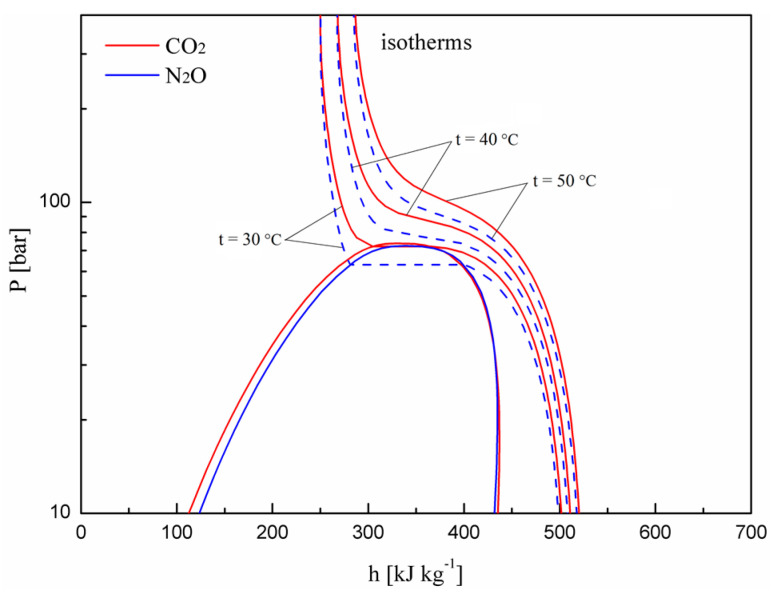
Pressure–enthalpy diagrams and isotherms of CO_2_ and N_2_O.

In the transcritical CO_2_ cycle, the COP is significantly influenced by the discharge pressure [20,23]. An optimum discharge pressure exists that leads to the maximum COP for a given gas cooler exit temperature and evaporating temperature. The same situation exists in the transcritical N_2_O cycle. The existence of an optimum discharge pressure is due to the S-shaped isotherm in the supercritical region of the working fluid. Figure 3 shows the isotherms of CO_2_ and N_2_O at different temperatures.

In this study, the performances of the transcritical N_2_O and CO_2_ cycles have been evaluated on the basis of the maximum cooling COP corresponding to an optimum discharge pressure. The optimum cooling COP has been estimated for various operating conditions with a 1 bar step increase in discharge pressure. Specific exergy losses in each component for different cycles are calculated and compared under the same operating conditions and at the optimum discharge pressures.

### 4.1. Energy Analysis of the Throttling Valve Cycle and Expander Cycle Using N_2_O

**Figure 4:**
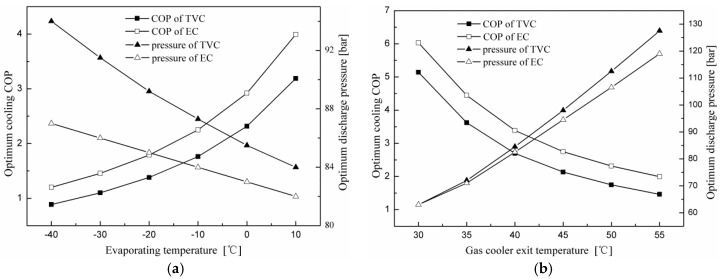
Effects of the evaporating and gas cooler exit temperatures on the performance of the two cycles. (**a**) Effect of the evaporating temperature on system performance; (**b**) effect of the gas cooler exit temperature on system performance.

The variations in the maximum cooling COP and corresponding optimum discharge pressure with evaporating and gas cooler exit temperatures for the throttling valve cycle and expander cycle using N_2_O are shown in Figure 4. It can be found that the variation trends of the expander cycle are similar to those of the throttling valve cycle. Results show that with a decrease in gas cooler exit temperature and an increase in evaporating temperature, the cooling COPs of the two cycles increase and the corresponding optimum discharge pressures decrease. Figure 4 also shows that the performance of the expander cycle is superior to the throttling valve cycle in terms of a higher cooling COP and lower discharge pressure at the same operating conditions. The COP improvement of the expander cycle is due to smaller expansion losses caused by the high pressure difference compared with the throttling valve cycle.

### 4.2. Energy Analysis of the Expander Cycle Using N_2_O and CO_2_ as Refrigerants

**Figure 5:**
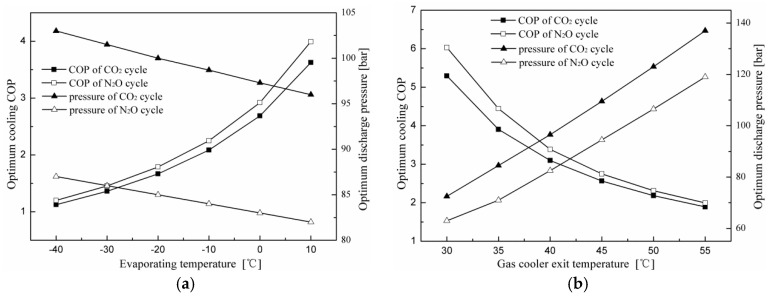
Effects of the evaporating and gas cooler exit temperatures on the performance of the two cycles. (**a**) Effect of the evaporating temperature on system performance; (**b**) effect of the gas cooler exit temperature on system performance.

The variations in the maximum cooling COP and corresponding optimum discharge pressure with evaporating and gas cooler exit temperatures for the expander cycle using N_2_O and CO_2_ are shown in Figure 5. With an increase in evaporating temperature and a decrease in gas cooler exit temperature, the cooling COP increases sharply and the corresponding optimum discharge pressure decreases gradually. It can be seen that the N_2_O expander cycle has a lower optimal discharge pressure and higher optimum cooling COP compared to the expander cycle using CO_2_ at the same operating conditions. The optimal discharge pressure for the N_2_O cycle is lower than that of the CO_2_ cycle due to the dissimilar nature of the isotherms. Figure 3 shows the different isotherms of CO_2_ and N_2_O. It can be seen that N_2_O has a lower pressure corresponding to the smallest slope of the isotherm than CO_2_ at the same temperature. The COP improvement of the N_2_O cycle is due to the greater cooling capacity and smaller power consumption by the compressor compared with the CO_2_ cycle.

**Figure 6:**
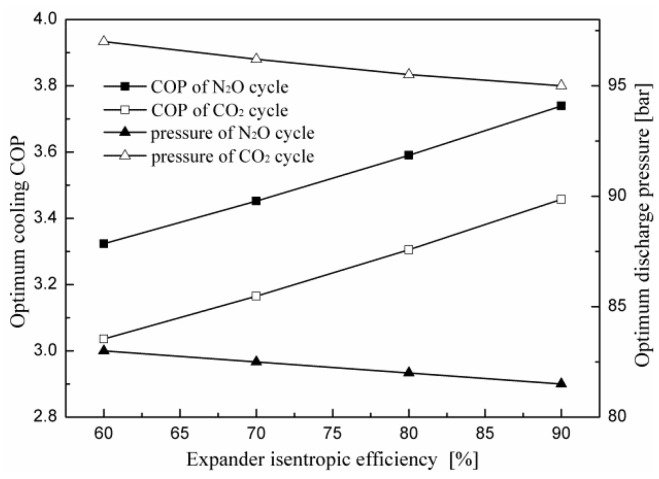
Effect of expander isentropic efficiency on the performance of the two cycles.

The variations in the optimum cooling COP and corresponding optimum discharge pressure with the isentropic efficiency of the expander for the two cycles are shown in Figure 6. It is found that the increased isentropic efficiency of the expander results in a significant improvement in system performance while having a positive influence on the optimum discharge pressure. For an evaporating temperature of 5 °C and a gas cooler exit temperature of 40 °C, with an increase in isentropic efficiency from 60 to 90%, the cooling COP of the transcritical N_2_O cycle increases by 12.52% and the optimum discharge pressure decreases by 1.8%. The COP improvement of the cycle is due to a decrease in power consumption by the compressor. As the isentropic efficiency of the expander increases, more work is recovered from the expander, which offsets the compressor power consumption for the electric motor drives.

### 4.3. Exergy Analysis of the Throttling Valve Cycle and Expander Cycle Using N_2_O

**Figure 7:**
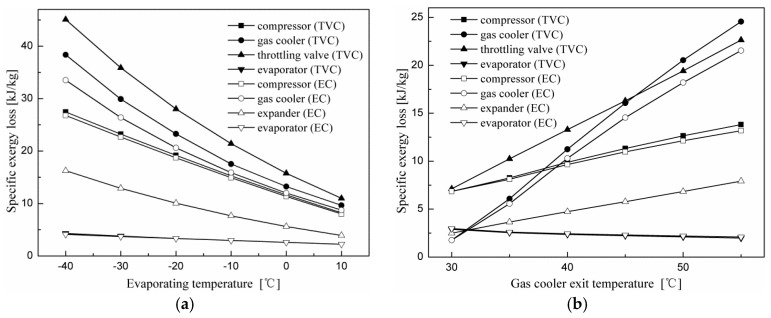
Effects of the evaporating and gas cooler exit temperatures on the specific exergy loss of each component for the throttling valve cycle and expander cycle. (**a**) Effect of the evaporating temperature on specific exergy loss; (**b**) effect of the gas cooler exit temperature on specific exergy loss.

Figure 7 shows variations in the specific exergy loss for each component with evaporating and gas cooler exit temperatures. It can be seen in Figure 7a that exergy losses of all components for the two cycles decrease with increasing evaporating temperature. It is shown in Section 4.1 and Section 4.2 that as the evaporating temperature increases, the optimum discharge pressure decreases for all cycles, and an increase in the evaporating temperature leads to an increase in suction pressure. When the discharge pressure decreases, the compressor discharge temperature is also reduced. Thus, the irreversibility decrease in the compressor is due to the fact that the compression ratio and the temperature difference between the inlet and the outlet are both reduced when the evaporating temperature increases. The irreversibility decrease in the gas cooler is due to a decrease in the discharge pressure and the temperature difference between the compressor discharge temperature and the gas cooler exit temperature.

Figure 7b shows that exergy losses of the system components of the two cycles, except for the evaporator, increase with gas cooler exit temperature. As the gas cooler exit temperature increases, the optimum discharge pressure increases for all cycles. When the suction pressure is constant, an increase in the discharge pressure results in an increase in the compressor discharge temperature and the compression ratio. Therefore, the irreversibility increase in the compressor is due to an increase in the compression ratio and the temperature difference between the inlet and outlet of the compressor. The irreversibility increase in the gas cooler is due to an increase in the discharge pressure and temperature difference between the compressor discharge temperature and the gas cooler exit temperature. In addition, it was found that the component-specific exergy loss in the N_2_O throttling valve cycle is greater than that of the corresponding component in the N_2_O expander cycle, except for the evaporator. The specific exergy losses of the evaporator for both cycles are almost the same at all operating conditions.

**Figure 8:**
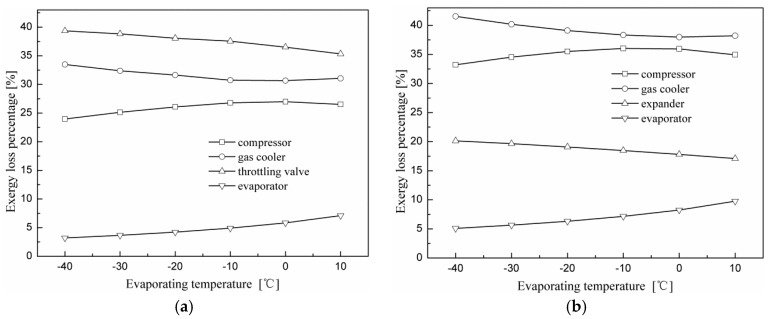
Effect of the evaporating temperature on the percentage of exergy loss for each component for the two cycles with the gas cooler exit temperature at 40 °C and at the optimal discharge pressure. (**a**) Throttling valve cycle; (**b**) expander cycle.

Figure 8 shows the percentage of exergy loss attributed to each component of the two N_2_O cycles versus various evaporating temperatures when the gas cooler exit temperature is 40 °C and at the optimal discharge pressure. The largest exergy loss for the N_2_O throttling valve cycle occurs in the throttling valve, which accounts for 38% of the total exergy loss. This is due to the large throttling losses caused by a large pressure difference during the expansion process. The exergy losses of the gas cooler and compressor are ordered second and third, which account for 32% and 25%, respectively. The evaporator accounts for about 5% of the total exergy loss. Different from the throttling valve cycle, the largest exergy loss of the expander cycle occurs in the gas cooler, which accounts for 38% of the total exergy loss. The exergy losses of the compressor and expander are ordered second and third, which account for 35 and 20%, respectively.

**Figure 9:**
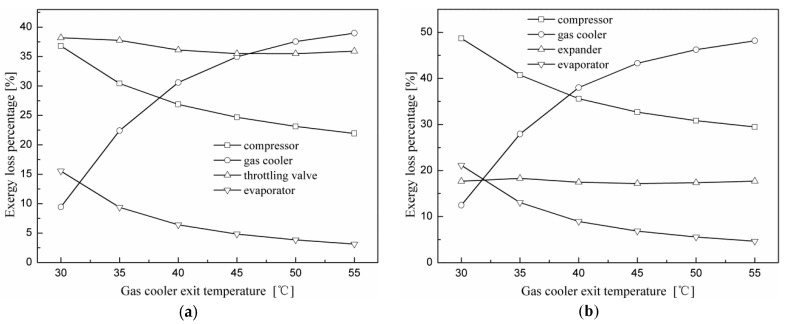
Effect of the gas cooler exit temperature on the percentage of exergy loss of each component for the two cycles with the evaporating temperature at 5 °C and at the optimal discharge pressure. (**a**) Throttling valve cycle; (**b**) expander cycle.

Figure 9 shows the percentage of exergy loss attributed to each component of the two N_2_O cycles versus various gas cooler exit temperatures when the evaporating temperature is 5 °C and at the optimum discharge pressure. It can be observed that along with the growth of the gas cooler exit temperature, exergy loss percentages of the compressor and evaporator for both cycles decrease gradually, and that of the gas cooler increases significantly. In addition, the exergy loss percentages of the throttling valve and the expander vary slightly. It can be found by comparing Figure 8 and Figure 9 that the variation in component exergy loss is more sensitive to the gas cooler exit temperature than to the evaporating temperature.

**Figure 10:**
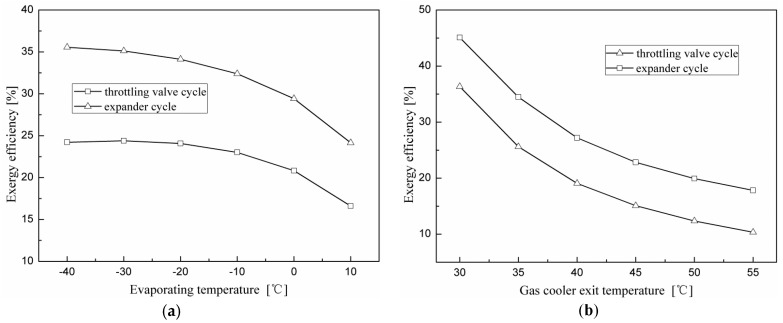
Effects of the evaporating and gas cooler exit temperatures on exergy efficiency for the throttling valve cycle and expander cycle. (**a**) Effect of the evaporating temperature on exergy efficiency; (**b**) effect of the gas cooler exit temperature on exergy efficiency.

Figure 10 depicts the exergy efficiencies of the investigated cycles as functions of the evaporating and gas cooler exit temperatures. It can be seen that the exergy efficiency decreases with an increase in the evaporating and gas cooler exit temperatures. It can be inferred from Figure 7a that as the evaporating temperature increases the exergy losses of all components for the two cycles decrease. Although the total exergy loss decreased, the phenomenon that the exergy efficiency decreases with an increase in the evaporating temperature is due to the reduction in the power consumption of the compressor. The decrease in the exergy efficiency with the gas cooler exit temperature is due to an increase in the discharge pressure and temperature difference between the compressor discharge temperature and the gas cooler exit temperature. It can also be seen from Figure 10b that the N_2_O expander cycle performs better than the throttling valve cycle and gives an average 46% increase in exergy efficiency. Different from the variation trend of exergy efficiency, variations in the COPs for the two cycles increase with the evaporating temperature. This means that the maximum COP and the maximum exergy efficiency cannot be obtained at the same time for the investigated cycles.

### 4.4. Exergy Analysis of the Expander Cycle Using N_2_O and CO_2_ as Refrigerants

**Figure 11:**
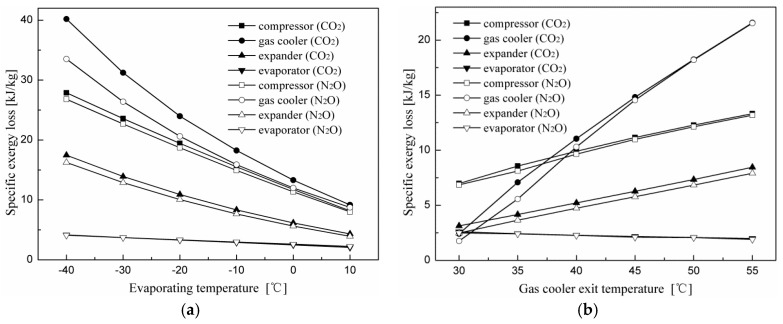
Effects of the evaporating and gas cooler exit temperatures on specific exergy loss of each component for the expander cycle using N_2_O and CO_2_ as refrigerants. (**a**) Effect of the evaporating temperature on specific exergy loss; (**b**) effect of the gas cooler exit temperature on specific exergy loss.

Figure 11 indicates specific exergy loss change trends of each component for the expander cycle using N_2_O and CO_2_ as the refrigerants with evaporating and gas cooler exit temperatures. It can be seen that the component irreversibility of the CO_2_ expander cycle is greater than that of the corresponding component in the N_2_O expander cycle, except for the evaporator. The specific exergy loss difference for corresponding components of the two cycles decreases with increased evaporating and gas cooler exit temperatures.

**Figure 12:**
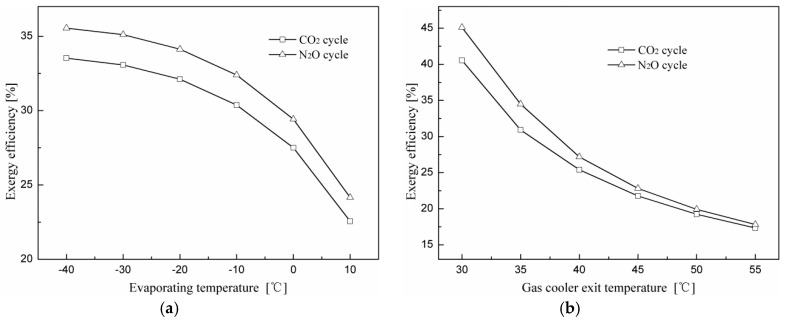
Effects of the evaporating and gas cooler exit temperatures on exergy efficiency for the expander cycles using N_2_O and CO_2_ as refrigerants. (**a**) Effect of the evaporating temperature on exergy efficiency; (**b**) effect of the gas cooler exit temperature on exergy efficiency.

Figure 12 shows exergy efficiency variations of the expander cycle using N_2_O and CO_2_ as refrigerants at different evaporating and gas cooler exit temperatures. The N_2_O expander cycle performs better than the CO_2_ expander cycle and gives an average 6.5% increase in exergy efficiency within the range investigated. In addition, both exergy efficiencies of the two cycles decrease with an increase in the evaporating and gas cooler exit temperatures.

**Figure 13:**
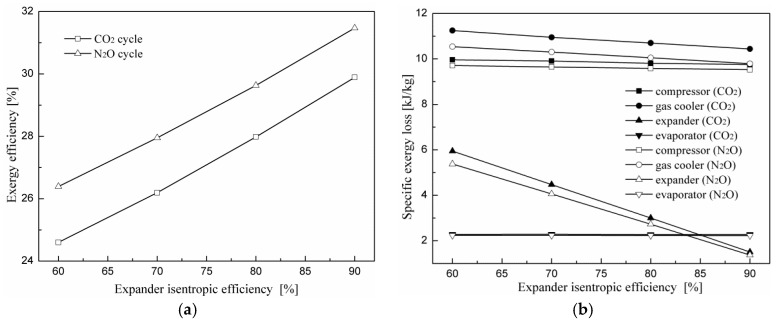
Effect of expander isentropic efficiency on exergy efficiency and the specific exergy loss of each component for expander cycles using N_2_O and CO_2_ as refrigerants. (**a**) Effect of expander isentropic efficiency on exergy efficiency; (**b**) effect of expander isentropic efficiency on specific exergy loss.

Figure 13 indicates exergy efficiency and specific exergy loss change trends of each component for the expander cycle using N_2_O and CO_2_ as the refrigerants with expander isentropic efficiency at a gas cooler exit temperature of 40 °C and an evaporating temperature of 5 °C. It can be seen that the exergy efficiencies of the two cycles increase with the expander isentropic efficiency. The exergy efficiency of the N_2_O expander cycle is on average 6.3% greater than that of the CO_2_ expander cycle for the same operating conditions. It can be seen from Figure 13b that all exergy losses in the system components have almost no change with expander isentropic efficiency, except for the expander. The specific exergy loss of the expander decreases gradually with the addition of expander isentropic efficiency.

## 5. Conclusions

Analyses based on the first and second laws of thermodynamics have been performed for a transcritical N_2_O refrigeration cycle with a throttling valve or with an expander, and thermal performances have been compared with those of a transcritical CO_2_ cycle. System models have been developed and the results were obtained when the gas cooler exit temperature was varied from 30 to 55 °C and the evaporating temperature varied from −40 to 10 °C. Based on our results, the following conclusions can be drawn:

(1) For the two cycles of the investigation, the optimum COP increases and the optimum discharge pressure decreases with an increase in the evaporating temperature and a decrease in the gas cooler exit temperature for both working fluids.
(2) Effects of the evaporating and gas cooler exit temperatures on the cooling COP and the corresponding optimum discharge pressures are similar for the N_2_O and CO_2_ expander cycles. The N_2_O expander cycle exhibits a larger COP than the CO_2_ expander cycle. In addition, the N_2_O system is safer than the CO_2_ system owing to a lower discharge pressure.
(3) At the given conditions, the N_2_O expander cycle has a lower optimum discharge pressure and a higher maximum cooling COP compared to the N_2_O throttling valve cycle.
(4) In the N_2_O throttling valve cycle, the irreversibility of the throttling valve is a maximum, and the exergy losses of the gas cooler and compressor are ordered second and third, respectively. In the N_2_O expander cycle, the largest exergy loss occurs in the gas cooler, followed by the compressor and the expander.
(5) Compared with the CO_2_ expander cycle and N_2_O throttling valve cycle, the N_2_O expander cycle has the smallest component specific exergy loss and the highest exergy efficiency. In addition, the maximum COP and the optimum exergy efficiency cannot be obtained simultaneously.

Therefore, replacement of the throttling valve with an expander in the transcritical N_2_O cycle can increase the cooling COP and exergy efficiency. The results provide the theoretical basis for optimizing cycle design and operational control of the transcritical nitrous oxide cycle with an expander. In the future, studies should be conducted on trade-offs between operating and capital costs of replacing throttling valves with expanders in transcritical refrigeration cycles.

## Figures and Tables

**Table 1:** Comparison of the properties of nitrous oxide (N_2_O) and carbon dioxide (CO_2_) [3].

Properties	N_2_O	CO_2_
ODP	0	0
GWP	240	1
Critical temperature (°C)	36.4	31.1
Critical pressure (bar)	72.5	73.84
Toxicity (ppm)	1000	5000
Triple point temperature (°C)	−90.82	−56.56
Molecular weight (g/mol)	44.013	44.01

**Table 2:** Energy balances for the throttling valve cycle and the expander cycle.

Subsystems	Throttling Valve Cycle	Expander Cycle
Compressor	*w_com_ = h_2_ − h_1_* *η_com,is_* *= (h_2s_ − h_1_)/(h_2_ − h_1_)*	*w_com_ = h_2_ − h_1_* *η_com,is_* *= (h_2s_ − h_1_)/(h_2_ − h_1_)*
Gas cooler	*q_gc_ = h_3_ − h_2_*	*q_gc_ = h_3_ − h_2_*
Expansion device	*h_3_ = h_4h_*	*w_exp_ = (h_3_ − h_4s_)* *η_exp,is_* *η_exp,is_* *= (h_3_ − h_4_)/(h_3_ − h_4s_)* *w_exp,com_ = w_exp_* *η_mt_*
Evaporator	*q_eva_ = h_1_ − h_4h_*	*q_eva_ = h_1_ − h_4_*
COP	*COP = q_eva_/w_com_*	*COP = q_eva_/w_com,elec_*

**Table 3:** Exergy loss for the components of the throttling valve and the expander cycles.

Subsystems	Throttling Valve Cycle	Expander Cycle
Compressor	*I_com_ = T_0_ (s_2−_s_1_)*	*I_com_ = T_0_ (s_2−_s_1_)*
Gas cooler	*I_gc_ = h_2_-h_3_-T_0_ (s_2−_s_3_)*	*I_gc_ = h_2_-h_3_-T_0_ (s_2−_s_3_)*
Expansion device	*I_tv_ = T_0_ (s_4h−_s_3_)*	*I_exp_ = T_0_ (s_4−_s_3_)*
Evaporator	*I_eva_ = T_0_ (s_1−_s_4h_) + (h_4h_ − h_1_) T_0_/T_r_*	*I_eva_ = T_0_ (s_1−_s_4_) + (h_4_ − h_1_) T_0_/T_r_*

## Abbreviation

**Nomenclature**	
COP	coefficient of performance (-)
TVC	throttling valve cycle (-)
ODP	ozone depletion potential (-)
GWP	global warming potential (-)
EC	expander cycle (-)
*h*	specific enthalpy (kJ kg^−1^)
*s*	specific entropy (kJ kg^−1^ K^−1^)
*w*	specific work (kJ kg^−1^)
*I*	specific irreversibility (kJ kg^−1^)
*P*	pressure (bar)
*T,t*	temperature (K, °C)
*q*	specific heat transfer rate (kJ kg^−1^)
**Greek Symbols**	
*η*	efficiency (%)
*ε*	exergy efficiency (%)
**Subscripts**	
0	reference environment
exp	expander
tv	throttling valve
is	isentropic process
com	compressor
eva	evaporator
gc	gas cooler
tot	total
r	refrigerated object
elec	electric
mt	mechanical transmission
